# Silver Nanoparticles with Mebeverine in IBS Treatment: DFT Analysis, Spasmolytic, and Anti-Inflammatory Effects

**DOI:** 10.3390/pharmaceutics17050561

**Published:** 2025-04-24

**Authors:** Mihaela Stoyanova, Miglena Milusheva, Vera Gledacheva, Mina Todorova, Nikoleta Kircheva, Silvia Angelova, Iliyana Stefanova, Mina Pencheva, Bela Vasileva, Kamelia Hristova-Panusheva, Natalia Krasteva, George Miloshev, Yulian Tumbarski, Milena Georgieva, Stoyanka Nikolova

**Affiliations:** 1 Department of Organic Chemistry, Faculty of Chemistry, University of Plovdiv, 4000 Plovdiv, Bulgaria; stoyanova@uni-plovdiv.bg (M.S.); or miglena.milusheva@mu-plovdiv.bg (M.M.); minatodorova@uni-plovdiv.bg (M.T.); 2 Department of Bioorganic Chemistry, Faculty of Pharmacy, Medical University of Plovdiv, 4002 Plovdiv, Bulgaria; 3 Department of Medical Physics and Biophysics, Faculty of Pharmacy, Medical University of Plovdiv, 4002 Plovdiv, Bulgaria; vera.gledacheva@mu-plovdiv.bg (V.G.); iliyana.stefanova@mu-plovdiv.bg (I.S.); mina.pencheva@mu-plovdiv.bg (M.P.); 4 Institute of Optical Materials and Technologies “Acad. J. Malinowski”, Bulgarian Academy of Sciences, 1113 Sofia, Bulgaria; nkircheva@iomt.bas.bg (N.K.); sea@iomt.bas.bg (S.A.); 5 University of Chemical Technology and Metallurgy, 1756 Sofia, Bulgaria; 6 Laboratory of Molecular Genetics, Epigenetics and Longevity, Institute of Molecular Biology “R. Tsanev”, Bulgarian Academy of Sciences, 1113 Sofia, Bulgaria; byvasileva@gmail.com (B.V.); karamolbiol@gmail.com (G.M.); milenageorgy@gmail.com (M.G.); 7 Institute of Biophysics and Biomedical Engineering, Bulgarian Academy of Sciences, 1113 Sofia, Bulgaria; kameliahristova@abv.bg (K.H.-P.); natalia.krasteva@yahoo.com (N.K.); 8 Department of Microbiology and Biotechnology, Technological Faculty, University of Food Technologies, 4002 Plovdiv, Bulgaria; tumbarski@abv.bg

**Keywords:** inflammatory bowel disease (IBD), gastrointestinal inflammation (GI), silver nanoparticles, drug delivery (DD), mebeverine hydrochloride (MBH), molecular docking, spasmolytic, anti-inflammatory activity, HepG2 cells

## Abstract

**Background/Objectives**: Mebeverine hydrochloride (MBH) is an antispasmodic agent used to regulate bowel movements and relax intestinal smooth muscle, but its application is limited by specific side effects; therefore, this study investigates the effects of previously synthesized MBH-loaded silver nanoparticles (AgNPs) on smooth muscle contractile activity and their anti-inflammatory potential as an alternative delivery system. **Methods:** The interactions of AgNPs with cholinergic inhibitors, selective antagonists, Ca^2+^ blockers, and key neurotransmitters were analyzed. In vitro, albumin denaturation suppression and ex vivo assays evaluated the anti-inflammatory effects of AgNPs-MBH, validated using a DFT in silico approach. To comprehensively assess the systemic impact and IBS treatment potential of AgNPs-MBH, we also examined in vitro their antimicrobial activity and hepatic cell responses, as the liver is a key organ in evaluating the overall safety and efficacy of nanoparticles. Additionally, the drug-release capabilities of Ag NPs were established. **Results**: Our findings indicate that AgNPs with MBH do not affect blocked cholinergic receptors, but their effects are more pronounced and distinct in amplitude and character than MBH. MBH-loaded AgNPs showed a lower anti-inflammatory effect than MBH but were still better than diclofenac. They also affected hepatic cell morphology and proliferation, suggesting potential for enhanced therapeutic efficacy. Drug-loaded AgNPs are considered not bactericidal. **Conclusions**: Based on our results, drug-loaded AgNPs might be a promising medication delivery system for MBH and a useful treatment option for IBS. Future in vivo and preclinical experiments will contribute to the establishment of drug-loaded AgNPs in IBS treatment.

## 1. Introduction

Inflammatory bowel disease (IBD) is characterized by chronic gastrointestinal inflammation (GI) [1,2]. The condition affects between 5% and 10% of otherwise healthy individuals at any one point in time and, in most people, runs a relapsing and remitting course [3]. The occurrence of IBD in developing regions like Asia, Eastern Europe, and Africa has risen rapidly in recent years [4]. IBD is characterized as multifactorial, involving genetic susceptibility, immune dysfunction due to environmental factors (diet, chemicals, stress), and microbial exposure [5,6,7,8], with an imbalance in gut microbiota [6,9,10,11].

Although current treatments for IBD offer certain therapeutic benefits, their efficacy is often constrained by non-specific drug distribution, gastrointestinal metabolism, and notable side effects [12]. A well-designed drug delivery (DD) system can enhance therapeutic outcomes by employing controlled drug release, prodrugs, pH-responsive mechanisms, and micro- or nanoparticulate formulations [13,14]. Several pharmacological agents—including corticosteroids, antibiotics, and immunosuppressants—have recently demonstrated effectiveness in promoting mucosal healing. However, there remains a pressing need for innovative biomaterial-based platforms capable of delivering drugs more efficiently and with reduced adverse effects. Such platforms can serve as targeted DD systems, facilitating colon-specific delivery and ensuring sustained drug release at the injury site [15]. Although micro- and nanoscale DD strategies can potentially prolong colonic retention and offer additional therapeutic advantages for IBD, the accumulation of micro- and nanoparticles in inflamed intestinal tissues is size-dependent [16,17], underscoring their promise as leading candidates for regulated drug delivery.

Nanoparticles (NPs) of noble metals have gained considerable attention over the past few decades due to their expanding applications in pharmaceutical and biomedical fields, including DD, photothermal therapy, radiotherapy, and imaging [18]. Among these, gold and silver nanoparticles have been the most extensively synthesized, attributed to their remarkable biological activity and unique physicochemical properties [19,20,21]. A major challenge in utilizing silver nanoparticles (AgNPs) for DD—particularly in inflammatory bowel disease—is achieving precise, site-specific delivery to the colon [22,23].

The application of carbohydrates in NP synthesis has recently garnered significant interest. Various green synthesis approaches have been developed for producing nanosized AgNPs, such as starch-mediated biosynthesis [24,25,26], and other methods employing polyoxometalates, polysaccharides, and classical chemical reactions like Tollens’ and Fehling’s [27,28,29,30,31]. A wide range of mono-, di-, and polysaccharides have been employed as reducing and capping agents in the synthesis of noble metal NPs, owing to their sustainability, availability, low cost, non-toxicity, renewability, biodegradability, and excellent biocompatibility [32,33,34,35,36].

**Figure 1:**
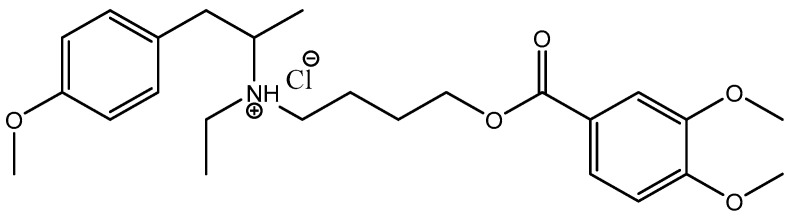
Chemical structure of mebeverine hydrochloride (MBH).

Our recent work synthesized AgNPs functionalized with mebeverine hydrochloride (MBH) and assessed their cytotoxic and genotoxic properties [37]. To date, few strategies for MBH targeting have been reported, including MBH-loaded electrospun nanofibers [38], mucoadhesive buccal tablets for local anesthesia [39], and molecularly imprinted polymer-based electrochemical sensors for MBH detection [40]. MBH is an antispasmodic drug (Figure 1) that can potentially exert a local anesthetic effect via blocking voltage-gated Na^+^ channels. As a musculotropic antispasmodic agent, MBH directly influences colonic muscle activity [41]. Due to its muscle-relaxing properties, MBH is widely used to treat GI spasmodic disorders such as abdominal pain, intestinal disturbances, and bowel discomfort [42].

Unlike the mentioned and already reported molecules, when depositing MBH into NPs, a change in the spasmolytic activity of the drug-loaded AgNPs is expected, both in terms of the pharmacological profile and in terms of the speed of action and the duration of the effect [43,44,45,46]. Therefore, a plausible first step in the assessment of various characteristics that play a key role in the drug (ligand)/AgNP recognition process is calculations based on Density Functional Theory (DFT) [47,48,49]. These simulations provide valuable information about the electronic structure and physicochemical properties and the change of these properties when the drug is adsorbed on the nanoparticle’s surface [50]. Integrating in silico calculations with experimental validation is crucial for retrieving solid evidence of the potential therapeutic application and probable mechanism of action. Advancements in computational and experimental methodologies will continue to enhance our understanding of AgNPs, paving the way for their safe and practical application in various fields.

The primary aim of this study is to investigate the specific effects of MBH-loaded silver nanoparticles (AgNPs) on smooth muscle (SM) contractility compared to the effects of free MBH. To achieve this, we explored the interactions of drug-loaded AgNPs with key regulators of SM function, including cholinergic inhibitors (atropine, ipratropium), selective muscarinic antagonists (pirenzepine, gallamine, 4-DAMP), calcium channel blockers (hexamethonium, decamethonium), and neurotransmitters (acetylcholine and 5-HT). These experiments were complemented by in silico modeling to validate the predicted antispasmodic properties of the conjugates. Additionally, we evaluated the anti-inflammatory potential of MBH-loaded AgNPs through albumin denaturation and ex vivo assays. We investigated their impact on hepatic cell morphology and proliferation to assess their safety and systemic effects. By integrating pharmacodynamic, anti-inflammatory, and cytotoxicity profiles, this study aims to comprehensively assess MBH-loaded AgNPs as a novel and targeted therapeutic strategy for irritable bowel syndrome (IBS).

## 2. Materials and Methods

### 2.1. Synthetic Protocol

MBH or 4-[ethyl-[1-(4-methoxyphenyl)propan-2-yl]amino]butyl 3,4-dimethoxybenzoate hydrochloride is a crystalline white powder with a molecular weight of 429.5 g/mol. The free base of mebeverine is essentially insoluble in water and ethanol, but its hydrochloride salt is very soluble in water and freely soluble in alcohol [51,52]. With a pKa of around 10 and a plasma half-life of 2.5 h, it requires frequent administration because of its short half-life [53]. MBH (Duspatalin) is marketed as a 200 mg extended-release capsule with a recommended dosage of two times per day. Its musculotropic antispasmodic activity, which directly affects the smooth muscles of the GI tract, is utilized. It is used effectively in treating IBS, a condition marked by abdominal pain and irregular bowel movements.

The NMR data was completely consistent with data reported in the literature [54]. HRMS electrospray ionization (ESI) *m*/*z* calcd. for [M + H]^+^ C_25_H_36_O_5_N^+^ = 430.25880, found 430.25788 (mass error ∆m = −2.14 ppm).

**Figure 2:**
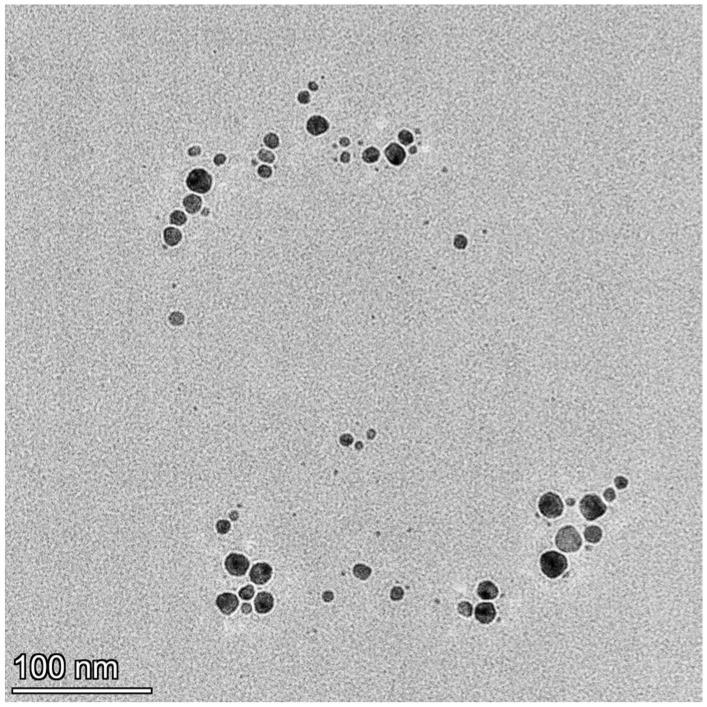
TEM visualization of drug-loaded silver nanoparticles with MBH.

Drug-loaded AgNPs were synthesized according to previously reported methods [37]. Briefly, 1.25 g (0.007 mol) of fructose was dissolved in 25 mL of water and refluxed for 2 min; then, 0.63 mL of 0.01 M AgNO_3_ solution was added. MBH was added to the solution at a concentration of 1 mg/mL. After roughly 5 min of reflux, the solution’s color changed to a light yellow, signifying the formation of AgNPs. The synthesized AgNPs were characterized using different analytical techniques, such as UV-Vis, Transmission electron microscopy (TEM, Talos 1.15.3, Thermo Fisher Scientific, Waltham, MA, USA), dynamic light scattering (DLS, Brookhaven BI-200 goniometer, Brookhaven Instruments Corporation, Nashua, NH, USA), and the zeta potential (Brookhaven BI-200 goniometer, Brookhaven Instruments Corporation, Nashua, NH, USA). The TEM images confirmed the synthesis of smaller spherical particles up to 95 nm in size (Figure 2). The drug-loaded AgNPs were obtained with a controllable size due to carbohydrate assistance in the presence of [Ag(NH_3_)_2_]^+^ [55]. The carbohydrate coating also decreases the agglomeration rate and size [56].

The zeta potential of drug-loaded AgNPs was −19.19 mV. The negative charge of the zeta potential refers to the carboxylic groups of carboxylic acid obtained when Ag^+^ is reduced to Ag°. Carboxylic acids, obtained in the oxidation of sugars, provide a negative surface charge density to counteract the van der Waals forces responsible for particle coalescence [30,31,57].

### 2.2. DFT Calculations

An initial step in the current research presents the theoretical assessment of the process of drug adsorption on the surface of AgNPs coated with carbohydrate molecules (e.g., fructose) and the further evaluation of the change of some specific physicochemical characteristics deriving from the HOMO-LUMO differences. For this purpose, the Density Functional Theory approach was utilized in the Gaussian 09 Suite of Programs [58]. The structures of the drug under study, MBH, in its two non-charged and cationic forms, and a simplified model for the carbohydrate surface comprising three fructose molecules, as well as the resulting complexes, were subjected to complete geometry optimization at the B3LYP level of theory in conjunction with the triple zeta 6-311++G(d,p) basis set in the gas phase found most suitable for the investigated systems in our previous research [59]. The lack of negative frequencies in the vibrational frequency analysis indicated a local minimum on the potential energy surface. Hence, following thermodynamics, ref. [60] the adsorption energy, ΔE_ads_, can be found as follows:

ΔE_ads_ = ΔE_ads_ (products) − ΔE_ads_ (reactants),

where the electronic energies obtained during optimization were implemented in the equation. For a more proper representation, thermal energies, including zero-point energy, Eth, were further retrieved and included in an equation for calculating the enthalpy, ΔH. The overall change in the enthalpy could then be computed as follows:

ΔH = ΔE_ads_ + ΔE_th_ (products) − ΔE_th_ (reagents) + PΔV,

where PΔV stands for a work term that accounts for the change in the number of molecules during a reaction; hence, a negative value of ΔE_ads_/ΔH indicates the successful adsorption of MBH on the carbohydrate layer, whereas a positive one suggests an improbable reaction.

### 2.3. Cell Culturing Conditions and IC_50_ Determination for AgNPs with MBH Treatments

HepG2 cells, originating from human liver carcinoma, were purchased from the European Collection of Authenticated Cell Cultures (ECACC) and maintained in Dulbecco’s Modified Eagle Medium (DMEM) supplemented with 10% fetal bovine serum (FBS; Sigma-Aldrich, Darmstadt, Germany). The cells were incubated at 37 °C in a humidified atmosphere containing 5% CO_2_. A solution of 0.05% trypsin and 0.02% EDTA (Sigma-Aldrich, Germany) was used to detach cells for subsequent experiments. HepG2 cells were seeded in 24-well plates at a density of 2.5 × 10^4^ cells per well for cytotoxicity assessments, including cell viability. After an initial 24-h incubation period, the cells were exposed to AgNPs, MBH, and their combined nano-formulation for designated time points. Cell viability and metabolic activity were assessed using the WST-1 assay. After 24- and 72-h incubation periods with the tested compounds, the medium was removed, and the cells were rinsed with PBS. A fresh medium containing a WST-1 reagent (10% *v*/*v*) was added to each well, followed by a 1-h incubation in the dark. Absorbance was measured at 450 nm using a Thermo Scientific Multiskan Spectrum ELISA reader (Thermo Scientific, Tokyo, Japan). GraphPad software (GraphPad Prism, version 10.0.3; GraphPad Software, San Diego, CA, USA) calculated the half-maximal inhibitory concentrations (IC_50_) based on dose-response curves.

### 2.4. Fluorescence-Activated Cell Sorting (FACS) Analysis of HepG2 Cells

#### Cell Cycle Analysis and Cellular Morphology

Flow cytometry (BD FACSCalibur™ Flow Cytometer; Becton Dickinson & Company, Franklin Lakes, NJ, USA) was used to assess cell cycle progression and morphological changes in HepG2 cells treated with MBH, AgNPs, and AgNPs with MBH at IC_50_ concentrations for 24 and 72 h. The cells were fixed in 76% cold ethanol and stored at −20 °C for 24 h. Following fixation, the cells were centrifuged, washed with PBS, and incubated with 100 µg/mL RNase A at 37 °C for 30 min. Propidium iodide (PI) staining was performed using a 50 µg/mL solution for 30 min in the dark. Fifty thousand events were recorded per sample using flow cytometry with the excitation at 488 nm, and the data were analyzed using FlowJo™ software (Version 10, Becton Dickinson, Franklin Lakes, NJ, USA).

### 2.5. Ex Vivo Spasmolytic Activity 

The study was performed on the gastric SM of male Wistar rats weighing 270–290 g. Three or four muscle strips were taken from a rat’s stomach. The number of muscle preparations used for each data point is indicated by n. The isolated SM preparation was placed upright in a thermostated vessel for isolated organs that was 15 mL in volume and oxygenated with physiological fluid at a temperature of 37 °C. The SM preparations consisted of circular dissections, 12–13 mm in length and 1.0–1.1 mm in width, which were used to record isometric contractile activity. All the transducers used in our experiments were made by the Tissue Organ Bath System (159920 Radnoti, Dublin, Ireland) according to a previously described procedure [59,61].

### 2.6. In Vitro Inhibition of Albumin’s Denaturation

The denaturation inhibition was performed following Milusheva et al.’s method [61]. The reaction mixture contained 0.2 mL of the tested AgNPs in DMSO (0.5 mg/mL) and 0.5 mL of a 5% aqueous solution of human albumin (Albunorm 20, Octapharma (IP) SPRL, 1070 Anderlecht, Belgium). For 15 min, the samples were incubated at 37 °C. Subsequently, each tube was filled with 2.5 mL of phosphate-buffered saline (pH 6.3), and the samples were heated at 80 °C for 30 min before being cooled for 5 min. The turbidity was measured at 660 nm spectrophotometrically (Cary 60 UV-Vis, Agilent Technologies, Santa Clara, CA, USA). The percentage of the inhibition of protein denaturation was calculated as follows:

percentage of inhibition of denaturation = [(A_control_ − A_AgNPs_)/A_control_] × 100.

The control represents 100% protein denaturation. The commercially available anti-inflammatory drugs diclofenac and acetylsalicylic acid (ASA) were used for comparison. Additionally, MBH controls were made. Their anti-inflammatory effect was determined as a percentage of the inhibition of albumin denaturation, following the same protocol.

### 2.7. Immunohistochemistry

The sections (5 µm thick) obtained from rat stomachs were deparaffinized and subjected to the immunohistochemical analysis using the monoclonal antibody IL-1β (E-AB-52153) (Elabscience Biotechnology Inc., Houston, TX, USA) and the polyclonal antibody 5HT3, 1:300 (E-AB-32268), (Ellabscience Biotechnology Inc., Houston, TX, USA) [62].

All the microphotographs were taken using a Leica DM1000 LED microscope (Leica Microsystems, Wetzlar, Germany) and an ICC50W digital camera (Leica Microsystems, Wetzlar, Germany).

### 2.8. Morphometric Analysis

The immune response intensity in the SM cells was quantified in arbitrary units (AUs) on sections immunostained for IL-1β and 5HT3 receptors. The average pixel intensity was determined using specialized software, ranging from 0 (black) to 256 (white). Measurements were performed on microphotographs of the SM cells at ×200 magnification, with at least 50 points assessed per slice. A total of five slices were analyzed for each animal.

### 2.9. In Vitro Drug Release

In vitro drug release was evaluated using the dialysis bag approach. A dialysis membrane was hydrated in distilled water for 24 h (MWCO 12 kDa, Sigma-Aldrich, St. Louis, MO, USA). Drug-loaded Ag NPs were dispersed in 10 mL of phosphate-buffered saline (PBS), transferred to the dialysis bag, and closed with a plastic clamp. Each bag was placed into a beaker with 40 mL of PBS (dialysis medium, pH 7.4). Aliquots with 4 mL of each dialysis medium were taken for measurements, and then the fresh medium was added at specified intervals. The drug release experiment lasted 130 h. The mean results of triplicate measurements and standard deviations were reported. Additionally, drug-only controls were made.

### 2.10. Antimicrobial Assay

The agar diffusion method evaluated the antimicrobial activity of drug-loaded AgNPs against various microorganisms. The tested microorganisms included Gram-positive bacteria, such as *Enterococcus faecalis*, *Staphylococcus aureus*, *Listeria monocytogenes*, *Bacillus subtilis*, and *Bacillus amyloliquefaciens*; Gram-negative bacteria, including *Escherichia coli*, *Salmonella typhimurium*, *Pseudomonas aeruginosa*, *Proteus vulgaris* G, and *Klebsiella pneumoniae*; two yeasts, *Candida albicans* and *Saccharomyces cerevisiae*; and six fungi (*Aspergillus niger*, *Aspergillus flavus*, *Penicillium* sp., *Rhizopus* sp., *Mucor* sp. (plant isolate), and *Fusarium moniliforme*) from the collection of the Department of Microbiology at the University of Food Technologies—Plovdiv, Bulgaria.

The procedure involved spreading a suspension of each test microorganism (10^6^ cfu/cm^3^) onto specific nutrient agar media. Next, 6 mm diameter wells were created in the agar, and 60 μL of the tested substance solution (1 mg/cm^3^) was added to each well. The Petri dishes were then incubated at appropriate temperatures (37 °C for bacteria and *C. albicans* and 30 °C for *S. cerevisiae*) for 24–48 h. The fungi *A. niger*, *A. flavus*, *Penicillium* sp., *Rhizopus* sp., *Mucor* sp., and *F. moniliforme* were grown on Malt Extract Agar at 30 °C for 7 days or until sporulation. After incubation, the inhibition zones around each well were measured, with zones more significant than 7 mm considered inhibition zones. Each test was performed in triplicate, and the results were reported as mean values of the inhibition zone diameters.

The antimicrobial activity was determined by measuring the diameter of the inhibition zones around the wells on the 24 h and 48 h of incubation. The tested microorganisms with inhibition zones of 18 mm or more were considered sensitive; those in which the zones were from 12 to 18 mm were considered moderately sensitive; and those in which the inhibition zones were up to 12 mm or completely missing were considered resistant [63].

### 2.11. Statistical Analysis

The SPSS 23.0 program (SPSS Inc., Chicago, IL, USA) was used for the statistical analysis. All the data are expressed as the mean ± SD (standard deviation). The number of tissue preparations used in each experiment is indicated by n. The statistical significance of the two independent groups was analyzed using an independent sample *t*-test. The level of statistical significance was considered as *p* < 0.05.

## 3. Results and Discussion

### 3.1. DFT Analysis in AgNP Evaluation

**Figure 3:**
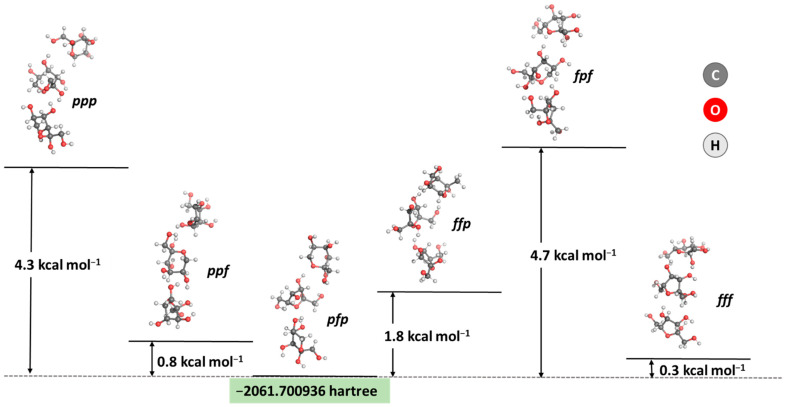
Optimized geometries and obtained energy differences between six combinations of the two most stable ring forms of fructose in solution (in kcal mol^−1^). The five-membered cycle is denoted as *f*, whereas the six-membered ring is denoted as *p*.

As the first step in the current research, an analysis of the most energetically favorable composition of the carbohydrate layer in the AgNPs was performed. A combination of three fructose molecules was modeled, as suggested in a previous study [59], following the computational restriction regarding the size of the studied system. Fructose is known to exist primarily in two forms: as a furanose (denoted as *f*) and a pyranose (denoted as *p*), with five- and six-membered rings, respectively [64]. Hence, six combinations between these forms were modeled, and their stability was assessed. The results obtained are presented in Figure 3.

**Figure 4:**
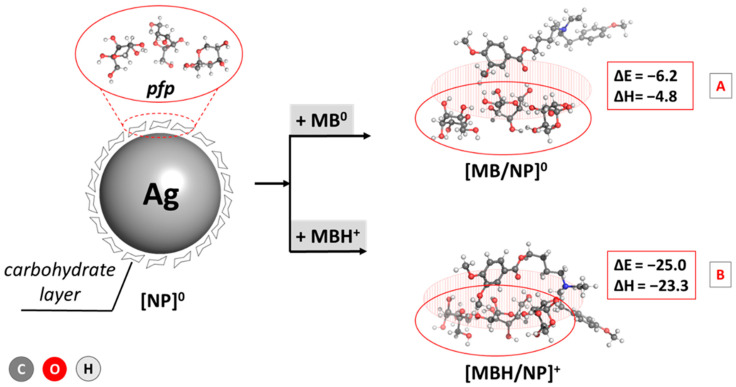
Adsorption energies and enthalpies of MBH/AgNP complex formation (in kcal mol^−1^) for the drug in the [A] neutral form and the [B] cationic form. The structures are optimized at the B3LYP/6-311++G(d,p) level.

The calculations prove the most thermodynamically favorable composition of three fructose molecules: the pyranose/furanose/pyranose combination appears to be the most stable configuration, closely followed by the trifuranose and pyranose/pyranose/furanose combinations, which differ by only 0.3 and 0.8 kcal mol^−1^ in stability, respectively. These results strongly correspond to experimental data claiming that fructose exists in a solution’s 2:1 pyranose:furanose ratio [64]. Therefore, this particular composition for a simplified model of the carbohydrate outer layer in the nanoparticle was considered when further assessing the interaction with the MBH molecule. The energy and enthalpy for the absorption of MBH on the carbohydrate-coated surface of a silver nanoparticle were calculated and are depicted in Figure 4.

The performed computations indicate an energetically justified reaction of drug-loaded AgNP formation as the observed changes of ΔE_ads_ and enthalpy, ΔH, are strongly negative: −6.2/−4.8 and −25.0/−23.3 kcal mol^−1^ for the interaction between the carbohydrate layer and MBH in neutral/cationic form, respectively. Due to the positive charge of the ligand in the second reaction, however, the complexation appears much more favorable than a reaction for the neutral form, with an energy gain that is up to about 18 kcal mol^−1^ greater in the former case. This outcome could be explained by the stronger interaction between the charged amino group from the drug and the HO-groups from the fructose ring being ion–dipole in nature as opposed to the weaker dipole–dipole interaction in the case of a neutral MBH. Still, the calculations provide strong evidence for successful MBH adsorption on AgNPs regardless of the charge of the drug.

Further interpretation of the obtained results includes the frontier molecular orbitals energies, an indispensable parameter explaining the electronic behavior of the studied structures. Data regarding the energies of the highest occupied (HOMO)/lowest unoccupied (LUMO) molecular orbitals, along with the quantum chemical descriptors connected to them, are presented in Table 1.

The energy gap, ΔE, accounts for a molecule’s chemical reactivity and stability: the more significant the gap, the more reactive but less stable a compound is. Hence, the adsorption of MBH in its neutral form onto the surface of the carbohydrate layer promotes its reactivity but slightly lowers its stability. In contrast, this trend reverses in the case of a cation. Nevertheless, both the bare ligands and their complexes with fructose appear chemically stable and do not disintegrate to their primal elements, as evidenced by the negative value of the chemical potential Pi. Furthermore, the hard–soft–acid–base approximation states that soft compounds tend to interact with molecules of a biological origin more efficiently [65], which suggests that among the studied structures, drug-loaded AgNP^0^ and MBH^+^ would be more prone to interaction with proteins as their chemical softness, ó, is more significant compared to the rest of the compounds. The high electrophilicity index of MBH^+^ outlines it as the best electron density acceptor with an estimated ù of 16.04 eV, which is an expected result when considering its positive charge. The adsorption on the fructose layer lowers this value to 14.28 eV for the drug-loaded AgNP^+^ complex. This descriptor is much lower for the non-charged MBH and the corresponding drug-loaded AgNP^0^ structure, estimated at 5.52/6.05 eV, suggesting that these compounds would be prone to supplying electrons. Overall, the provided DFT analysis of the structures under study draws a clear picture of the adsorption process of an MBH molecule (neutral and cationic) on the surface of a fructose-coated silver nanoparticle and is correlated with its physicochemical parameters of great significance and hence justifies further experiments. Note that the mechanism of therapeutic action of MBH corresponding to binding in the two active centers of albumin have already been subjected to investigation in our previous work [66] and will not be discussed any further.

### 3.2. Evaluation of Ex Vivo Spasmolytic Effect

**Figure 5:**
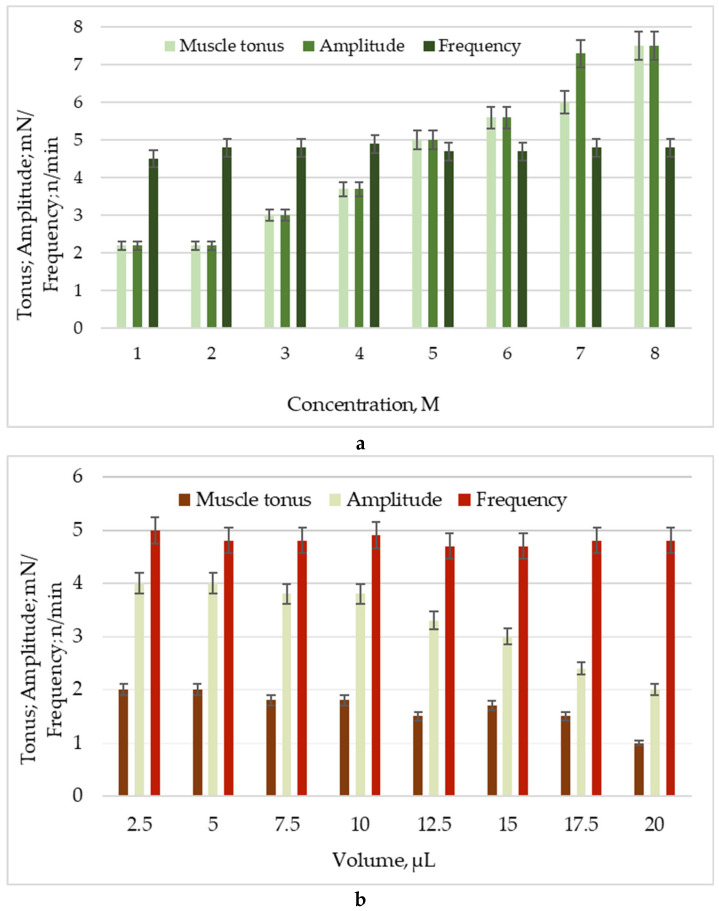
Changes in main parameters of spontaneous contractile activity: muscle tonus (mN), amplitude (mN), and frequency (n/min) under the influence of: (**a**) MBH; (**b**) AgNPs with MBH.

It is common practice to investigate the drug concentration–response relationship using both in vitro and ex vivo models. The degree of influence can be ascertained by isometrically measuring concentration-dependent changes in the mechanokinetic parameters of spontaneous contractions after the tested chemical has been applied to the model system. To track target molecules’ activating or inhibiting effects as modulators of muscle activity, we employed an isolated SM model from a rat’s stomach in this investigation. After pretesting the isolated SM samples for viability with ACh, we determined the following details using the known cumulative dose-dependent effects of MBH and its deposition into AgNPs. MBH caused a sharp contractile reaction with dose-dependent changes in the tonic response and a statistically significant increase in the contraction amplitude (Figure 5a). AgNPs with MBH, on the other hand, exhibited mild tonic relaxation and a 50% reduction in the amplitude of spontaneous contraction. Neither substance induced statistically significant changes in the frequency of contractile activity (Figure 5b).

The observed differences point out different mechanisms of action on the cellular processes underlying SM’s spontaneous reactions. At least three different mechanisms are involved in the polyvalent spasmolytic effects, which are known to be not specific to any one system. These mechanisms include competitive antimuscarinic activity, direct musculotropic action, including Ca^2+^ ion exchange, and local anesthetic action resulting from inhibiting norepinephrine uptake [67].

On the other hand, the single independent application of AgNPs does not alter the SM tonus in isolated SM from the trachea. However, the simultaneous application of AgNPs and ACh results in a slight increase in ACh-induced SM contractility, after which the muscle tone does not return to the baseline level. The authors of these reported findings attribute the observed effects to increased nitric oxide production [68].

To clarify the pathway of influence of the drug-loaded AgNPs in SM cellular mechanisms, we applied two of the primary neurotransmitters—ACh and 5HT [69,70]. After cholinergic or serotonergic stimulation, SM preparations respond with an initial sharp increase component (phasic contraction) due to the increased level of ionized cytosolic Ca^2+^. This is followed by a stable tonic (sustained) contraction caused by extracellular Ca^2+^ influx into SM cells [71].

This contractile activity pathway appears unaffected by MBH-loaded AgNPs, as the pre-incubation of tissues does not significantly alter the responses to the two neurotransmitters. Interestingly, MBH itself causes a remarkable 98% reduction. The likely explanation is the presence of nanoparticles.

The ex vivo tissue bath model allows for the independent or combined exogenous application of these substances, monitoring and assessing changes in the electrical, pharmacological, or electro-pharmacological responses of treated tissue segments. Due to the potential differences in their mechanisms of action, which could result in either the contraction or the relaxation of the SM, we further investigated the changes in the contractile response strength of the SM under the influence of AgNPs with MBH at a volume of 20 μL in the presence of blockers and activators of spontaneous contractility (Table 2). We found that the effects of AgNPs and MBH are not significantly changed when mAChRs are blocked by the non-selective receptor antagonist atropine (10^−6^ M, n = 6).

Additional research using specific and non-specific muscarinic and nicotinic acetylcholine receptor antagonists was conducted to better understand the mechanism of action of compounds encapsulated in Ag NPs. The responses to AgNPs with MBH were considerably changed by the use of specific antagonists, namely pirenzepine 10^−5^ M (M1 mAChR blocker), gallamine 10^−5^ M (M2 mAChR blocker), 4-DAMP 3 × 10^−7^ M (M3 mAChR blocker), hexamethonium 10^−5^ M (nAChR blocker), and decamethonium 10^−5^ M (nAChR blocker), to block muscarinic and nicotinic AChRs (Table 3).

Through this particular cellular mechanism, we attribute the effects to the capacity of MBH and AgNPs with MBH to activate mAChRs and nAChRs on SM cells, thereby regulating the critical mechanokinetic parameters of spontaneous contractions. Our findings demonstrated that when Ag NPs with MBH interact with specifically blocked cholinergic receptors, their effects are more noticeable and different in amplitude and character, while MBH has no effect.

In summary, we suggest that the biological activity of drug-loaded AgNPs is due to an underlying mechanism that directly connects mAChR and nAChR activation.

### 3.3. In Vitro Inhibition of Albumin Denaturation

Steroidal and nonsteroidal anti-inflammatory drugs (NSAIDs) treat inflammation, fever, and pain. The indications, mode of action, administration, adverse effects, and monitoring of NSAIDs are essential for healthcare professionals [72,73]. Anti-inflammatory drugs can prevent thermally induced protein denaturation in a dose-dependent manner [66,74].

**Figure 6:**
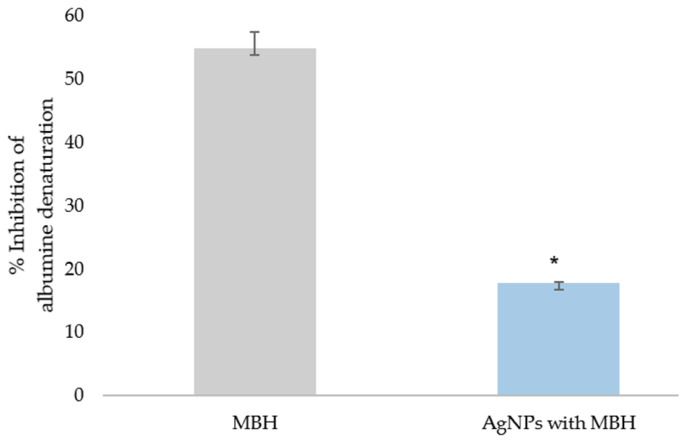
Inhibition of albumin’s thermal denaturation by MBH and drug-loaded AgNPs (%); * *p* < 0.05.

Our results for the in vitro anti-inflammatory activity of MBH and AgNPs with MBH were calculated as a percentage of the inhibition of albumin’s thermal denaturation (Figure 6). We found that MBH-loaded AgNPs exhibit three times lower activity than MBH (*p* < 0.05). The anti-inflammatory effect of MBH could be explained by the potential for attaching the albumin molecule via its functional groups [66]. The lesser anti-inflammatory effect of MBH-loaded AgNPs is most likely due to the interaction of MBH’s functional groups with the NPs.

**Figure 7:**
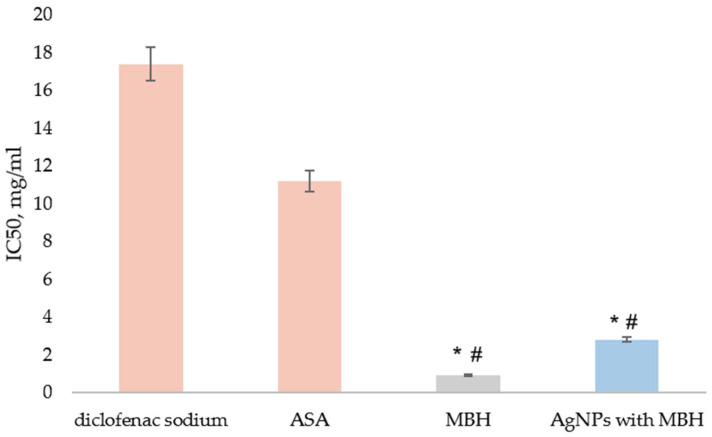
Inhibition of thermal denaturation of albumin by MBH (grey) and AgNPs with MBH (blue). The anti-inflammatory activity of the tested substances was compared to diclofenac’s and acetylsalicylic acid (ASA)’s activity (orange). The results are expressed as IC_50_, mg/mL. ^#^ *p* < 0.05 compared to diclofenac; * *p* < 0.05 compared to ASA.

We found that MBH and MBH-loaded AgNPs demonstrated 20-fold and 6-fold greater protection than diclofenac and 12-fold and 4-fold greater protection than ASA, respectively (*p* < 0.05) (Figure 7).

Further ex vivo experiments were carried out to verify these findings and assess the anti-inflammatory activity of AgNPs with MBH on nNOS and interleukin-1 (IL-1β) expression, compared to MBH.

### 3.4. Ex Vivo Anti-Inflammatory Activity

IBD and Crohn’s disease are characterized by the increased production of inflammatory chemicals by immune cells [75]. Proinflammatory cytokines, including IL-1, IL-6, and IL-17, are released in response to many disease conditions, including IBD, and significantly trigger intestinal inflammation [76]. Drug-loaded AgNPs provide great promise for local delivery and fewer side effects when used to treat IBS. One of the goals of selective delivery is to use nanoparticles to deliver drugs and accumulate them at the site of inflammation through immune cell absorption [77,78,79,80].

**Figure 8:**
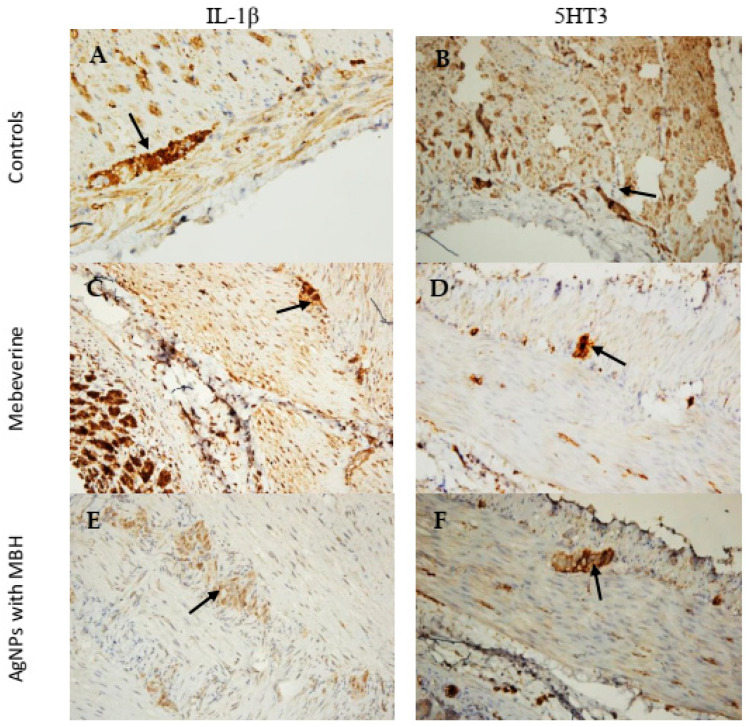
Microphotographs of SM preparations from the rat corpus incubated with MBH and MBH-loaded AgNPs for 1 h. (**A**) Control stained for IL-1β (black arrow), ×200. (**B**) Control stained for 5HT3 (black arrow), ×200. (**C**) SMs incubated with MBH showed the increased expression of IL-1β in the myenteric plexus (black arrow), ×200. (**E**) SMs incubated with AgNPs with MBH showed the weak expression of IL-1β in the myenteric plexus (black arrow), ×200. (**D**) SMs incubated with MBH, weakly stained cells in the myenteric plexus for 5HT3 (black arrow), ×200. (**F**) SMs incubated with AgNPs with MBH showed the weak expression of 5HT3 in the myenteric plexus (black arrow), ×200.

In SM cells incubated with MBH, the measured intensity and density of IL-1β expression (160.1 ± 5.4 AU) were comparable to those incubated with the controls (169.7 ± 8.9 AU, *p* < 0.001, Figure 8A,C) (Figure 8B). In contrast, AgNPs with MBH (134.8 ± 3.2 AU) showed the lower expression of IL-1β (Figure 8B,E).

We found that the SMs incubated with MBH (155.5 ± 7.7 AU) had a lower intensity of expression than the control preparations (166.4 ± 6.6 AU, Figure 8B,D, *p* < 0.001) in the 5HT3 comparative analysis. In contrast, the SMs incubated with AgNPs with MBH (123.3 ± 6.9 AU) had a weaker expression of 5HT3 in the myenteric plexus (Figure 8A,F).

To confirm the in vitro anti-inflammatory effect of AgNPs with MBH, we also measured ex vivo IL-1β and 5HT3 expression. The data analysis revealed varying degrees of expression. Compared to the control SMs and MBH, AgNPs significantly decreased the intensity of IL-1β expression and suppressed 5HT3 expression. Our results demonstrated that AgNPs exhibited better anti-inflammatory effects in terms of IL-1β expression when compared to the control samples. Drug-loaded AgNPs also showed a superior inhibitory effect on the 5-HT3 channel receptor. Our results align with previous research demonstrating that 5-HT3 receptor antagonists, including ramosetron and ondansetron, reduce intestinal damage [81,82].

The obtained AgNPs are essential for testing anti-inflammatory properties and their effect on serotonin receptors, particularly the 5-HT3 channel receptor. The synthesized AgNPs can also be used in drug delivery and applying monoamine neurotransmitters [83].

### 3.5. Cytotoxicity Assessment of AgNPs with MBH in HepG2 Cells

To evaluate the cytotoxic effects of MBH, AgNPs, and their combined formulation drug-loaded AgNPs, IC_50_ values were determined at 24- and 72-h post-treatment in HepG2 cells. The results indicated a time-dependent increase in IC_50_ values for all the tested formulations (Table 4). At 24 h, the IC_50_ values were as follows: MBH—17.16 µg/mL, AgNPs—6.35 µg/mL, and AgNPs-MBH—14.16 µg/mL. After 72 h, the IC_50_ values increased to 27.50 µg/mL for MBH, 17.79 µg/mL for AgNPs, and 17.94 µg/mL for AgNPs with MBH.

These findings suggest that AgNPs exhibited the highest cytotoxicity among the tested formulations, with the lowest IC_50_ values at both time points, which aligns with previous studies indicating the potent cytotoxic effects of AgNPs in hepatic cell lines [84]. The AgNPs-MBH combination demonstrated intermediate cytotoxicity, with slightly increased IC_50_ values at 72 h compared to 24 h, indicating potential cellular adaptation or reduced sensitivity over time. The observed increase in IC_50_ values over time for all compounds may indicate a cellular adaptation or reduced sensitivity to the treatments. This trend was noted in other studies where prolonged nanoparticle exposure resulted in increased IC_50_ values, possibly due to cellular defense mechanisms [85].

### 3.6. Fluorescence-Activated Cell Sorting (FACS) Analysis of HepG2 Cell Morphology and Granularity

**Figure 9:**
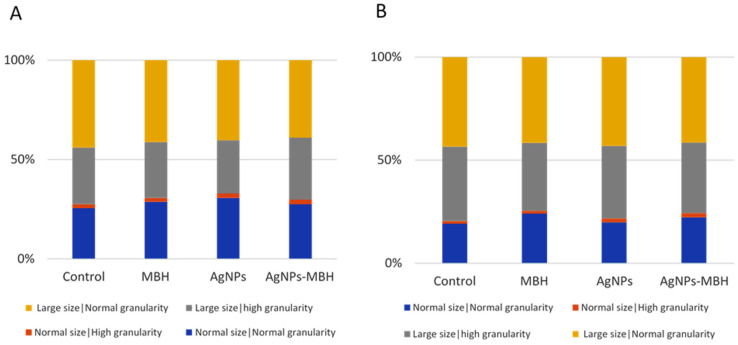
Effects of MBH, AgNPs, and their combination on HepG2 cell morphology and granularity at 24 h (**A**) and 72 h (**B**), assessed by FACS.

The fluorescence-activated cell sorting (FACS) analysis revealed distinct effects of MBH, AgNPs, and their combination on HepG2 cell morphology over time (Figure 9).

At 24 h, MBH, plain AgNPs and drug-loaded AgNPs increased the population of cells with standard size and granularity compared to the non-treated control cells. A minor rise in cells with high granularity was observed across all the treated groups. AgNPs reduced the proportion of larger cells with high granularity, whereas MBH and AgNPs with deposited MBH increased this population. The proportion of large cells with regular granularity remained relatively stable, with MBH slightly increasing and AgNPs reducing this population (Figure 9A). The relatively stable population of large cells with regular granularity under MBH treatment further supports its role in maintaining cellular integrity.

After 72 h, MBH maintained a higher number of normal-sized cells with regular granularity, and under AgNPs and drug-loaded AgNPs they were also increased (Figure 9B). The number of cells with high granularity was elevated under the AgNPs and AgNPs with MBH treatments compared to the control. Over time, AgNPs increased large cells with high granularity, while MBH and AgNPs-MBH led to a reduction, suggesting a potential cellular adaptation. In the population of large cells with regular granularity, AgNPs had the most pronounced increase, followed by MBH and drug-loaded AgNPs, indicating a time-dependent shift in cellular response.

These findings suggest that while MBH maintains a relatively stable cell distribution, AgNPs and their combination with MBH induce dynamic changes in cell size and granularity, possibly reflecting variations in cellular adaptation mechanisms.

### 3.7. The Fluorescence-Activated Cell Sorting (FACS) Analysis of the HepG2 Cell Cycle After 24 and 72 h of Treatment with MBH, AgNPs, and Their Combination

**Figure 10:**
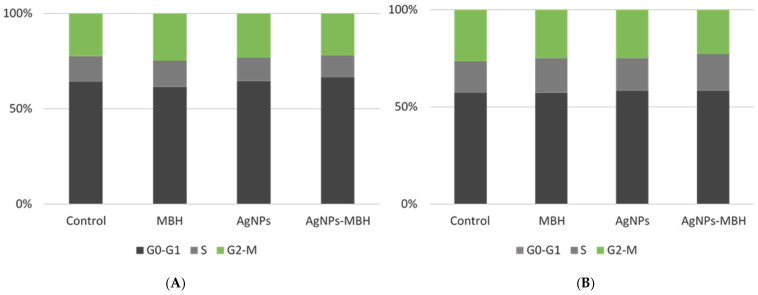
Effects of MBH, AgNPs, and their combination on HepG2 cell cycle at 24 h (**A**) and 72 h (**B**), assessed by FACS.

The fluorescence-activated cell sorting (FACS) analysis of the HepG2 cell cycle after 24 and 72 h of treatment revealed distinct effects of MBH, AgNPs, and their combination (Figure 10).

At 24 h, MBH slightly increased the proportion of cells in the G_0_-G_1_ phase compared to the control, suggesting a moderate delay in cell cycle progression. On the other hand, the AgNPs treatment led to a reduction in the G_0_-G_1_ phase, accompanied by decreases in both the S and G_2_-M phases (Figure 10A). This pattern may reflect cytotoxic effects or potential cell cycle arrest induced by AgNPs, highlighting their impact on cellular proliferation and viability [86,87,88]. The drug-loaded AgNPs resulted in a significant increase in the G_0_-G_1_ population, with a slight decrease in the S phase and a moderate increase in the G_2_-M phase. This indicates a potential synergistic effect, where the two agents together may promote a redistribution of cells across the different phases of the cell cycle.

At 72 h, the control cells showed a relatively stable distribution across all the phases. MBH treatment increased the proportion of cells in the S phase while slightly reducing G_2_-M-phase cells, indicating possible interference with DNA synthesis (Figure 10B). The AgNPs treatment led to an accumulation of cells in the G_0_-G_1_ and S phases, with a minor reduction in G_2_-M, suggesting a prolonged effect on cell cycle regulation. Interestingly, the drug-loaded AgNPs maintained a similar G_0_-G_1_-phase percentage to the control while increasing S-phase cells and decreasing G_2_-M-phase cells, potentially indicating a shift in the balance of proliferation and arrest mechanisms. The results indicate that while MBH and AgNPs impact cell cycle dynamics individually, their combination exerts unique regulatory effects that may modulate HepG2 cell proliferation. Further studies are warranted to elucidate the underlying mechanisms driving these effects and their potential implications for cancer treatment protocols.

### 3.8. Antimicrobial Activity

The AgNPs exhibit excellent antimicrobial activity, and this property can be beneficial, especially against microorganisms resistant to conventional antimicrobials [27]. The antibacterial activity of AgNPs against various bacteria results in the diverse inhibitory zone diameters reported recently [89]. The potential use of the antispasmodic mebeverine against *Staphylococcus aureus* was recently examined [90,91]. The authors demonstrated that mebeverine was beneficial in lowering the dosage of antibiotics and reducing the need for subsequent antibiotic administration to patients with various problems, including infection, spasm difficulties, and gastrointestinal ulcers.

Since MBH is administered orally in clinical settings, we anticipated that the drug-loaded AgNPs would also be taken orally. Considering that AgNPs have antibacterial properties, our next aim was to evaluate the antimicrobial effects of drug-loaded AgNPs and to determine whether they could disturb the balance of intestinal flora. The inhibition zones of bacterial and fungal growth caused by MBH-loaded AgNPs are outlined in Table 5.

Our experiments used six Gram-positive bacteria, six Gram-negative bacteria, two yeasts, and six fungi. The obtained results confirmed previously published results that MBH is bacteriostatic on some Gram-negative bacteria [27], but it was highly bactericidal when tested against *S. aureus*. We found that drug-loaded AgNPs exerted only modest activity against Gram-positive bacteria, including *Staphylococcus aureus* ATCC 25923, *Listeria monocytogenes* NBIMCC 8632, and *Enterococcus faecalis* ATCC 29212. Therefore, we can conclude that drug-loaded AgNPs are not bactericidal. To further assess the stability of drug-loaded AgNPs, their in vitro drug release was evaluated.

### 3.9. Drug Release

DD systems enter the stomach and small intestine via the mouth and then reach the colon. The nature and pH of gastric secretion and mucus influence drug release and absorption. To reach the colon intact, the DD system should bypass the stomach and small intestine barriers. GI transit varies from 1–3 h depending upon fasting or non-fasting periods [92].

In general, the small intestinal transit is not influenced by the physical state, size of the dosage form, and presence of food in the stomach. The mean transit time required for the dosage form to reach the ileocaecal junction is 3–4 h, and the time is inconsistent. During transit, the dosage form is exposed to enzymes (such as esterase, amylase, lipase, protease, nuclease, glycosidase, disaccharidases, etc.) in the small intestine. The bioavailability of drugs can be highly influenced by the colonic transit time. Compared with the other regions of the GI tract, the movement of material through the colon is slow and influenced by various factors, such as diet, dietary fibers, mobility, stress, disease conditions, and drugs. The colonic transit time of a capsule in an adult is 20–35 h. The release and absorption of the drug from a dosage form are governed by an improved residence time with a longer transit time and the contact of the dosage form with microflora in the colon [92].

Drug-release profiles from dialysis-based assays determine NP formulations’ in vivo–in vitro correlations and direct the development and quality control [93]. Under normal physiological conditions, the pH value from the stomach to the intestine showed an increasing trend, specifically, an acidic stomach (pH 1.5 to 3.5), duodenum (pH 6), terminal ileum (pH 7.4), terminal cecum (pH 6), and colon (pH 6.7) [94]. The drug-release study was carried out in a neutral medium (PBS, pH 7.4) to simulate the physiological conditions. A cellulose dialysis bag with a molecular weight cut-off of 12,400 Da was used to hold the solution of drug-containing Ag NPs. The pores in this cut-off were large enough to let the medication out of the dialysate while keeping the Ag NPs inside the membrane. Since the calibration curve for MBH was linear, it was possible to conduct the quantification tests.

The length of the intestine, the composition of the gut microbiota, individual variances in stomach and intestinal fluid, and peristalsis may all have a role in the transport time of the gastrointestinal system. There are considerable variations in gastrointestinal emptying periods, not just between healthy participants but also between IBD patients and healthy people, which may raise the uncertainty of when the medicine will reach the colon. Although the small intestine’s average transport time is thought to be 4 h, individual differences often range from 2 to 6 h [94]. Therefore, determining the drug release at the first 6 h was crucial for our investigation.

**Figure 11:**
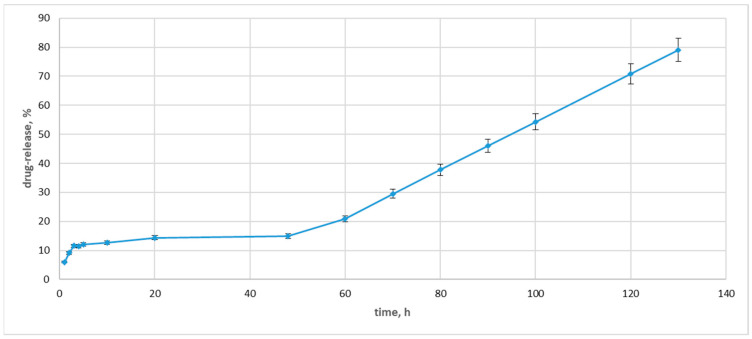
Drug-release concentrations for drug-loaded Ag NPs.

During the initial 5 to 20 h of the drug release experiment, the absorbance intensity rose with time, indicating an increasing concentration of the MBH in the dialysate (Figure 11).

After the 48 h, the drug release pattern changed drastically, with the concentration of the released MBH in the dialysate increasing by 14%. Then, the concentration of the released drug rose, and at the 130 h, it indicated an 80% drug release. The observed drug release was slower than those previously measured [59]. We can explain this drug release with the carbohydrate coating. The fructose loads MBH more effectively and slows down the drug release.

According to these preliminary findings, Ag NPs might be a helpful medication delivery mechanism for MBH and a potent tool for treating inflammatory bowel disease. For this reason, additional research on long-acting delivery formulations and potential medical uses for drug-loaded Ag NPs will also be required to add to the benefits of this study.

## 4. Conclusions

Our results provide valuable insights into the biological activity of the drug-loaded AgNPs studied from a spasmolytic and anti-inflammatory point of view.

Drug-loaded AgNPs exhibited mild tonic relaxation and a 50% reduction in the amplitude of spontaneous contraction. At the same time, MBH caused a sharp contractile reaction with dose-dependent changes in the tonic response and a statistically significant increase in the contraction amplitude. When drug-loaded AgNPs interact with specifically blocked cholinergic receptors, their effects are more noticeable and different in amplitude and character, while MBH has no effect. Therefore, the biological activity of drug-loaded AgNPs is due to an underlying mechanism that directly connects mAChR and nAChR activation.

In vitro and ex vivo experiments were conducted to further investigate the anti-inflammatory effect of drug-loaded AgNPs. In vitro, the results showed that MBH and drug-loaded AgNPs demonstrated better protection in albumin denaturation than known NSAIDs. The ex vivo experiments confirmed these results, demonstrating that AgNPs exhibited better anti-inflammatory effects regarding IL-1β expression and a superior inhibitory effect on the 5-HT3 channel receptor.

The provided DFT analysis confirmed the adsorption process of an MBH molecule (neutral and cationic) on the surface of fructose-coated AgNPs. It correlated with its physicochemical parameters of great significance, which justifies experiments.

Drug-loaded AgNPs were identified as not being bactericidal. Additionally, their in vitro drug release was also assessed. We found that fructose loads MBH more effectively and slows down the drug release.

Future in vivo and preclinical experiments will contribute to the establishment of drug-loaded AgNPs as a potential antispasmodic against inflammatory diseases in the GI tract.

Cytotoxicity results showed that plain AgNPs exhibited the most potent cytotoxicity among the tested formulations (AgNPs, MBH, and drug-loaded AgNPs) in HepG2 cells, with the lowest IC_50_ values at 24 and 72 h. The drug-loaded AgNPs showed intermediate cytotoxicity, with a slight increase in IC_50_ values over time, which could indicate cellular adaptation or reduced sensitivity to the treatment. The overall trend of increasing IC_50_ values over time for all the compounds points to possible cellular defense mechanisms or adaptation to prolonged nanoparticle exposure.

Our results demonstrate the distinct effects of MBH and AgNPs on HepG2 cell morphology over time. MBH appears to support stable cell distribution and maintain normal morphology, whereas AgNPs and drug-loaded AgNPs induce dynamic alterations, suggesting varied cellular adaptation responses. These findings are essential for refining nanoparticle-based drug delivery strategies.

The flow cytometry analysis further showed that MBH and AgNPs individually influence the HepG2 cell cycle, while their combination produces unique regulatory effects that may impact cellular proliferation. Investigating these interactions is vital for developing more effective therapeutic approaches. Additional studies are needed to uncover the mechanisms underlying these observations and their potential relevance to cancer treatment.

## Figures and Tables

**Table 1:** Quantum chemical descriptors obtained through DFT calculation at the B3LYP/6-311++G(d,p) level of theory: E_HOMO_, E_LUMO_, ΔE = E_LUMO_ − E_HOMO_, χ = −(E_HOMO_ + E_LUMO_)/2 (electronegativity), Pi = −χ (chemical potential), η = (E_LUMO_ − E_HOMO_)/2 (chemical hardness), σ = 1/η (chemical softness), and ω = Pi^2^/η (electrophilicity index). Data are presented in eV.

Compound	E_HOMO_	E_LUMO_	ÄE	÷	Pi	ç	ó	ù
MBH^0^	−5.71	−1.28	4.42	3.49	−3.49	2.21	0.45	5.52
MBH/AgNP^0^	−5.66	−1.46	4.20	3.56	−3.56	2.10	0.48	6.05
MBH^+^	−7.89	−3.70	4.19	5.80	−5.80	2.10	0.48	16.04
MBH/AgNP^+^	−8.28	−3.46	4.82	5.87	−5.87	2.41	0.41	14.28

**Table 2:** Changes in the strength of the contractile response of SM under the influence of MBH and AgNPs with MBH in the presence of the main neurotransmitters (ACh and 5-HT) and atropine (non-selective mAChR antagonist).

Compound	Individual Application, mN	Strength of the Contractile Reaction in the Background, mN	
		AgNPs with MBH	MBH
ACh	5.70 ± 0.23	4.98 ± 0.36	0.10 * ± 0.02
5HT	6.33 ± 0.45	6.17 ± 0.41	4.25 * ± 0.33
atropine	2.00 ± 0.18	2.10 ± 0.21	1.87 ± 0.18

The comparison is between spontaneous effects on CA in Krebs solution before and after the application of testing substances. All the data were expressed as the mean values ± standard error of the mean (mean ± SEM); n = 9; * statistically significant differences (*p* < 0.05).

**Table 3:** Changes in the strength of the contractile response of SM under the influence of AgNPs with MBH in the presence of a modulating agent of spontaneous contractility.

Compound	Individual Application	Strength of the Contractile Reaction in the Background of AgNPs with MBH, mN
Pirenzepine	1.16 ± 0.08	0.20 * ± 0.02
Gallamine	3.37 ± 0.21	1.22 * ± 0.12
4-DAMP	4.09 ± 0.40	1.08 * ± 0.07
Hexamethonium	3.78 ± 0.23	1.02 * ± 0.05
Decamethonium	2.55 ± 0.15	1.18 * ± 0.10

The comparison is between spontaneous effects on CA in Krebs solution before and after the application of testing substances. All the data were expressed as the mean values ± standard error of the mean (mean ± SEM); n = 6; * statistically significant differences (*p* < 0.05).

**Table 4:** Cytotoxicity (IC_50_) of MBH, AgNPs, and AgNPs-MBH in HepG2 cells at 24 and 72 h.

Samples	IC_50_ at 24 h [µg/mL]	IC_50_ at 72 h [µg/mL]
MBH	17.16	27.50
AgNPs	6.35	17.79
AgNPs-MBH	14.16	17.94

**Table 5:** Antimicrobial activity of drug-loaded AgNPs.

	*Bacillus subtilis* ATCC 6633	*Bacillus amyloliquefaciens* 4BCL-YT	*Staphylococcus aureus* ATCC 25923	*Listeria monocytogenes* NBIMCC 8632	*Enterococcus faecalis* ATCC 29212	*Salmonella typhimurium* NBIMCC 1672	*Escherichia coli* ATCC 25922	*Pseudomonas aeruginosa* ATCC 9027	*Aspergillus niger* ATCC 1015	*Aspergillus flavus*	*Penicillium chrysogenum*	*Fusarium moniliforme* ATCC 38932
Inhibition zone, mm	10 ± 0.0	10 ± 0.0	15 ± 0.2	15 ± 0.1	15 ± 0.5	12 ± 0.4	10 ± 0.1	12 ± 0.5	11 ± 0.0	8 ± 0.1	8 ± 0.0	11 ± 0.2

## Data Availability

The original contributions presented in the study are included in the article; further inquiries can be directed to the corresponding author.
